# The Efficacy of Humanized Antibody against the *Sporothrix* Antigen, gp70, in Promoting Phagocytosis and Reducing Disease Burden

**DOI:** 10.3389/fmicb.2017.00345

**Published:** 2017-03-03

**Authors:** José R. F. de Almeida, Karla L. Santiago, Gilberto H. Kaihami, Andrea Q. Maranhão, Marcelo de Macedo Brígido, Sandro R. de Almeida

**Affiliations:** ^1^Department of Clinical and Toxicological Analysis, Faculty of Pharmaceutical Sciences, University of São PauloSão Paulo, Brazil; ^2^Department of Biochemistry, Institute of Chemistry, University of São PauloSão Paulo, Brazil; ^3^Department of Cell Biology, Institute of Biological Sciences, University of BrasiliaBrasilia, Brazil

**Keywords:** humanized antibody, *S. schenckii*, sporotrichosis

## Abstract

Sporotrichosis is a subcutaneous mycosis distributed worldwide and is frequently reported in countries with tropical climates, as Latin America countries. We previously demonstrated that mice with sporotrichosis produce specific antibodies against a 70-kDa fungal protein, indicating that specific antibodies against this molecule may help to control the sporotrichosis. IgG1 monoclonal antibody was generated, and called mAbP6E7, in mice against a 70-kDa glycoprotein (gp70) of *S. schenckii*. The mAbP6E7 showed prophylactic and therapeutic activity against sporotrichosis. However, this antibody has a murine origin, and this can generate an immune response when administered to humans, precluding its use for a prolonged time. For its possible use in the treatment of human sporotrichosis, we humanized the mAbP6E7 by genetic engineering. Once expressed, the humanized antibodies had good stability and were able to bind to the 70-kDa cell wall antigens of *Sporothrix schenckii* and *S. brasiliensis*. The humanized P6E7 were able to opsonize *S. schenckii* yeasts, thus increasing the phagocytic index in human monocyte-derived macrophages. The treatment with humanized P6E7 decreased fungal burden *in vivo*. These data suggest that humanized P6E7 may have a therapeutic role in sporotrichosis.

## Introduction

Sporotrichosis is a subcutaneous mycosis, caused by *Sporothrix* sp., affecting animals and humans and frequently involves the lymphatic system. *Sporothrix* sp. include fungi with thermal dimorphism, in which the mycelial form, found in soil, is saprophytic, and the yeast form, which is found in the host tissue, is parasitic (Bonifaz and Vazquez-Gonzalez, 2010; Vasquez-del-Mercado et al., 2012). Sporotrichosis is distributed worldwide but is frequently reported in countries with subtropical and tropical climates, as the Latin America countries (Schechtman, 2010). The traumatic inoculation of the conidia and hyphae of this fungus results in the development of subcutaneous mycoses. Within the infected tissue, the fungus differentiates into its yeast form and may spread to other tissues (Barros et al., 2011). The largest epidemic of sporotrichosis because of zoonotic transmission was described in Rio de Janeiro between 1998 and 2004, in which 759 humans were diagnosed with sporotrichosis (Barros et al., 2008; Freitas et al., 2010). Marimon et al. (2007) described four new species, *S. globosa, S. brasiliensis, S. Mexicana*, and *S. luriei*. These new species have been found to have a worldwide distribution. However, *S. brasiliensis* is apparently restricted to Brazil, and *S. mexicana* is restricted to Mexico.

The fungus virulence and the host immune responses are essential for the development and severity of sporotrichosis. The recent sporotrichosis outbreaks in Rio de Janeiro city, demonstrates the emergence of feline zoonotic transmission and the prevalence of *S. brasiliensis* in the sporotrichosis, creating a new paradigm of this disease (Lopes-Bezerra and Mora-Montes, 2015).

Different drug protocols are used for the treatment of sporotrichosis and include terbinafine, potassium iodide, itraconazole, fluconazole, and the amphotericin B (Song et al., 2011). Adverse events, as vomiting and diarrhea, nausea, headache, hypersensitivity reactions, abdominal pain, and liver dysfunction, can occur (Lopez-Romero et al., 2011).

In a previous study, we demonstrated that sporotrichosis, caused by *Sporothrix schenckii* are able to produce specific IgG antibodies against a 70-kDa fungal protein (gp70), demonstrating that specific antibodies against the gp70 may help control the sporotrichosis (Nascimento et al., 2008). To understand the role of IgG mediated response in sporotrichosis, we produced an IgG1 monoclonal antibody against gp70 of *S. schenckii*, called mAbP6E7. To evaluate the protective effect of the mAbP6E7 *in vivo*, mice infected with *S. schenckii* were passively immunized. A significant reduction in fungal burden in the spleen and liver of mice treated with mAbP6E7 before and during experimental sporotrichosis caused by *S. schenckii* was observed (Nascimento et al., 2008). Recently, our group showed that mice treated with P6E7 and infected with a strain isolated from a case of feline sporotrichosis, strain 5110 (ATCC MYA – 4823) showed a decreased fungal burden in its organs (de Almeida et al., 2015).

The mAb P6E7 showed prophylactic and therapeutic activity against sporotrichosis. However, this antibody has a murine origin, and this can generate an immune response when administered to humans, precluding its use for a prolonged time. For its possible use in the treatment of human sporotrichosis, our proposal was the humanization of the mAb P6E7 through genetic engineering.

Here we humanized the mAbP6E7 and proved their binding ability to the fungus and effector function. We also demonstrated that the treatment with humanized P6E7 decreased the fungal burden in experimental sporotrichosis. Together, these data demonstrated the functionality of humanized P6E7 and their efficiency in the treatment of experimental sporotrichosis.

## Materials and Methods

### Microorganism and Culture Conditions

The strains used were *Sporothrix schenckii* M-64 (ATCC MYA 4822) and *Sporothrix brasiliensis* 5110 (ATCC MYA 4823). The strains were maintained by regular passages on animals and were grown on Sabouraud dextrose agar at 25°C for 7 days. The yeast form was obtained by cultivation in BHI agar at 37°C for 7 days.

### Animals

Female BALB/c mice at 10 weeks of age (weighing approximately 24 g) were obtained from the Animal House Production and Experimentation Facility of the Faculty of Pharmaceutical Sciences and Institute of Chemistry of the University of São Paulo. The mice were maintained in a SPF environment (specific pathogen free) and housed in temperature controlled rooms at 23–25°C with free access to food and water throughout the experiments.

### Human Ethics Statement

This study was carried out in accordance with the recommendations of Ethical Committee of the Faculty of Pharmacy of the University of São Paulo with written informed consent from all subjects. All subjects gave written informed consent in accordance with the Declaration of Helsinki.

### Animal Ethics Statement

This study was carried out in accordance with the recommendations of Guide for the Care and Use of Laboratory Animals of the National Institutes of Health. The protocol was approved by the Brazilian Conselho Nacional de Controle da Experimentação Animal (CONCEA).

### Computational Analysis

For computational analysis, a standard protocol was used (Silva et al., 2009). Briefly, human VH and VL framework sequences were extracted from the Swiss-Prot or GenBank. (A sequence search was performed with FASTA (Pearson, 2000) or BLAST (Altschul et al., 1997). ClustalW (Higgins et al., 1996) was used for multisequence alignment, running in BioEdit^1^. Sterically constrained atoms were detected using similar tri-dimensional models from the Protein Data Bank^2^. Model visualization and atom-to-atom distance calculations were performed in RASMOL version 2.6 (Bernstein, 2000). Variable region numbering followed Kabat’s convention (Kabat and Wu, 1991).

### Construction of Humanized FvFc Expression Vector

The plasmids were constructed using standard cloning methods (Sambrook and Russell, 2001). The VH and VL humanized P6E7 fragments were synthesized and confirmed by sequencing. The scFv (VH-linker-VL) region was cloned into a pMIRES vector using its *Xma*I and *Xho*I restriction sites to generate pHP6E7. This construction generated a VH-linker-VL fragment fused with the human IgG1 CH2CH3 domains. The nucleotide sequences were deposited in the GenBank with the accession numbers:

BankIt1984561 VL KY488459
BankIt1984561 VH KY488460

### hP6E7 Production and Purification

CHO-K1 cells were routinely cultured in Ham-F2 (HyClone) supplemented with 10% FBS. Approximately 9 × 10^6^ cells were seeded in a 15-cm cell culture dish. Cells were transfected using the jetPEI reagent (Polyplus Transfection) according to the manufacturer’s protocol and cultured in Ham-F2 with 1.25% ultra-low IgG FBS (Invitrogen). After 72 h, the culture supernatants were recovered. The supernatant containing the hP6E7 antibody were purified using a HiTrap Protein A column (GE Healthcare) equilibrated with 20 mM sodium phosphate at pH 7.0 and eluted with 0.1 M citric acid at pH 3.5. The eluted fractions were neutralized with 1 M Tris-HCl at pH 9.0. The buffer was exchanged using a PD-10 column (GE Healthcare) equilibrated with PBS. The proteins were concentrated with a Centriprep-Ultracel YM-10.000 MWCO (Millipore). The protein concentration was determined by BCA.

### Experimental Infection

Two groups of five mice each were inoculated through the intraperitoneal (i.p.) route with 5 × 10^6^ yeast cells of *S. schenckii* M-64 suspended in 0.1 mL of sterile PBS. After 3 days of infection, the treated groups received 100 μg of hP6E7 in 0.2 mL of sterile PBS. The control group was inoculated with 0.2 mL of sterile PBS. After 10 days of infection, the five mice of each group were euthanized in a CO_2_ chamber to measure the fungal burden. The spleen and liver were homogenized, and the supernatants were collected. The colony forming units (CFU) were assayed by serial dilution and plating on BHI agar plates. The plates were incubated up to seven days at 25°C. The CFU were counted, and the results are expressed as CFU per g of tissue.

### Human Monocyte-Derived Macrophages Isolation

Human monocyte-derived macrophages isolation was performed as previously described with a few modifications (Sousa et al., 2008). Briefly, the peripheral blood of healthy volunteers was collected in vacuum tubes containing heparin as an anticoagulant. The separation was performed by centrifugation for 40 min at 600 g on a density gradient using Ficoll–Hypaque (Sigma–Aldrich). The cells were aspirated and washed with sterile PBS. Positive selection using CD14^+^ magnetic beads was performed according to the manufacturer’s instructions (MiltenyiBiotec). The cells were centrifuged, washed and resuspended in sterile PBS. The monocytes were counted in a Neubauer chamber (2 × 10^5^) and plated on 24-well plates containing a round glass coverslip in the bottom in RPMI 1640 (Sigma–Aldrich) containing 10% FBS. The cell culture plates were incubated for seven days. Cells were left to adhere on culture plates for 2 days at 37°C, non-adherent cells were removed and the medium was exchanged for fresh RPMI 1640 containing 10% FBS.

### Phagocytosis Assay and Opsonization Condition

Prior to infection, *S. schenckii* M-64 yeast cells were opsonized with the hP6E7 antibody or not opsonized, as described previously, with some modifications (Nascimento et al., 2008). Briefly, 1 × 10^6^ *S. schenckii* M-64 yeast cells were incubated with 25 μg of the hP6E7 antibody or without it (control) in PBS for 1 h at 37°C under constant agitation. After this incubation, the yeast cells were washed three times with PBS to remove the unbound antibodies. Human monocyte-derived macrophages were infected with the opsonized or non-opsonized *S. schenckii* M-64 yeast cells (at a proportion of three yeast cells to 1 macrophage) for 5 h. The coverslips were stained with the hematological staining kit InstantProv (NewProv), and the phagocytic index (PI) was calculated according to the following formula:

$$\begin{array}{c} \mathrm{PI}\;=\;\left(\frac{\mathrm{Number}\;\mathrm{of}\;\mathrm{internalized}\;\mathrm{yeast}}{\mathrm{Number}\;\mathrm{of}\;\mathrm{Monocytes}\;\mathrm{in}\;\mathrm{the}\;\mathrm{same}\;\mathrm{field}}\right)\times 100 \end{array}$$

### Immunofluorescence

An immunofluorescence assay was performed as previously described with a few modifications (Nascimento et al., 2008). Briefly, *S. schenckii* M-64 yeast cells from 10-day-old cultures grown in BHI agar were collected and resuspended in sterile PBS. The yeast cells (5 × 10^6^) were incubated for 1 h in sterile PBS containing 0.2% gelatin. The cell suspension was then incubated for 2 h with 20 μg of hP6E7 diluted in PBS containing 0.2% gelatin. In the negative control, the yeast cells were incubated only with sterile PBS. After this period, the opsonized and non-opsonized yeast cells were washed three times in sterile PBS and incubated with the anti-human IgG antibody conjugated with Alexa Fluor 488 (Life Technologies) (1:200) in sterile PBS containing 0.2% gelatin. All steps were performed under agitation (37°C). The yeast cells were washed with sterile PBS and mounted on glass slides in Gelvatol. Then, they were examined with a Nikon Microphot FX microscope.

### Western Blot

The hP6E7 antibody was evaluated for its binding capacity against gp70, which is present in the cell wall of *S. schenckii* and *S. brasiliensis*. The proteins from cell wall were obtained from yeast cells of *S. brasiliensis* 5110 and *S. schenckii* grown in Erlenmeyer flasks containing 400 mL of YCG medium (yeast nitrogen–glucose casamino acids) for 7 days with constant agitation at 9 *g* at 37°C. Then, the yeast cells were collected and incubated with an extraction solution (2 mM DTT, 1 mM PMSF, and 5 mM EDTA in 25 mM Tris/HCl at pH 8.5) at 4°C for 2 h with mild agitation. The supernatant containing the cell surface proteins was collected and concentrated. Five micrograms of cell wall proteins was separated by electrophoresis using a 12% SDS polyacrylamide gel, and subsequently, the proteins were electrotransferred to Hybond ECL nitrocellulose membranes (GE Healthcare). Finally, the membranes were washed and developed using the ECL Plus Kit (GE Healthcare).

### Statistical Analysis

GraphPad Prism 5 (GraphPad Software) was used for all statistical analyses. The data were compared using the unpaired *t*-test. Differences were considered statistically significant at ^∗^*p* < 0.05; ^∗∗^*p* < 0.01; and ^∗∗∗^*p* < 0.001.

## Results

### Construction of the Humanized P6E7 Antibody as a scFc Molecule

Humanized antibodies are human-like antibodies in which only the complementarity determining regions (CDRs) are murine. This approach can be used to reduce the biggest issue with non-human antibody (HA) therapy, antibody immunogenicity (Clark, 2000). Therefore, to generate an effective antibody against the emerging mycosis sporotrichosis, the murine monoclonal antibody P6E7 was humanized. The P6E7 humanization was performed as described previously (Caldas et al., 2003). To reduce the mouse antibody immunogenicity, the VH and VL sequences of mAbP6E7 were compared to a HA database (GenBank, EMBL, PDB) to generate a similar human-like IgG polypeptide sequence.

**FIGURE 1:**
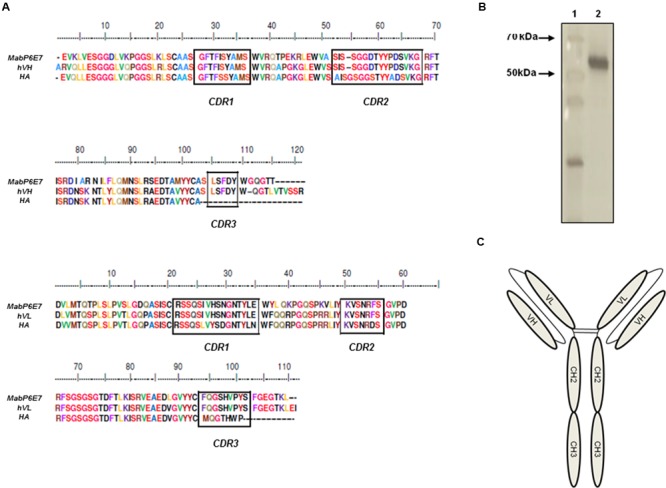
**Construction and purification of hP6E7. (A)** Multiple sequence alignment of MabP6E7, human antibody (HA), and humanized heavy chain (hVH) or humanized light chain (hVL). **(B)** hP6E7 was run under reducing condition in SDS-PAGE and stained with silver stain. Lane 1: Unstained protein molecular weight marker. Black arrows indicates 70 and 50 kDa. Lane 2: Purified hP6E7. **(C)** Schematic representation of a FvFc molecule. FvFcs are immunoglobulin-like structures comprising a homodimeric pair of VH and VL, joined with a flexible linker, a hinge, CH2, and CH3 domains. The nucleotide sequences were deposited in the GenBank with the accession numbers: BankIt1984561 VL KY488459 and BankIt1984561 VH KY488460.

These sequences were used as a framework for mAbP6E7 CDRs. Structural analyzes using the BioEdit Sequence Alignment Editor program were carried out to identify structural impediments in the human protein. The hP6E7 VH and VL were designed as scFc fragments (a single-chain Fv fused to a human IgG1 Fc) (**Figure 1C**) in the mammalian expression vector pMIRES. The sequences of heavy and light chain hP6E7 and MabP6E7 are similar (**Figure 1A**). The humanized antibody was expressed in the CHO-K1 cell line, and clones expressing the antibodies were obtained by positive selection. The humanized antibody was purified by affinity chromatography from cell culture supernatants. As expected, the hP6E7 preparation under reducing conditions is a 55-kDa homodimeric protein (**Figure 1B**), similar to other humanized scFc molecules (Silva et al., 2009).

### hP6E7 Recognizes the gp70 Protein

**FIGURE 2:**
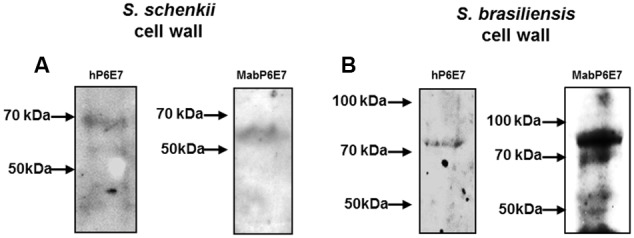
**hP6E7 binds to *Sporothrix* sp. antigens.** Western blot analysis of the cell wall extracts of *Sporothrix schenckii* **(A)** or *S. brasiliensis* **(B)**. Membrane was incubated with hP6E7 or mAbP6E7.

Our group characterized a 70-kDa antigen (gp70) in the exoantigen of *S. schenckii* and *S. brasiliensis* using mAbP6E7 (Nascimento et al., 2008; de Almeida et al., 2015). mAbP6E7 recognizes the gp70 protein from *S. schenckii* and *S. brasiliensis* in the exoantigen preparation of these strains (Castro et al., 2013). To evaluate the capacity of hP6E7 to bind to gp70, the cell wall preparation proteins from *S. schenckii* and *S. brasiliensis* were resolved by SDS-PAGE for the immunodetection of gp70 by hP6E7 in Western blot assays. We showed that hP6E7 and the mAbP6E7 binds proteins with the same molecular weights in *S. schenckii* (**Figure 2A**) and *S. brasiliensis* (**Figure 2B**). In *S. schenckii* and *S. brasiliensis* the molecular weight of the gp70 protein recognized by both antibodies was closer to 70 kDa.

Our group showed that the molecular weight of gp70 may vary due to glycosylation sites present in gp70 in *S. brasiliensis*, while these sites are absent in *S. schenckii* (Castro et al., 2013). In conclusion, we suggest that hP6E7 could recognize the gp70 antigen in both *Sporothrix* sp. species.

### hP6E7 Binds to the gp70 Protein on the Surface of *S. schenckii* and *S. brasiliensis*

**FIGURE 3:**
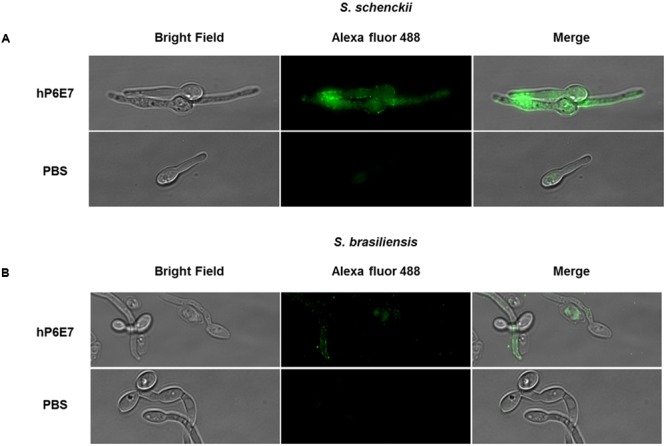
**hP6E7 binds to *S. schenckii* and *S. brasiliensis* cell wall. (A)** *S. schenckii* and **(B)** *S. brasiliensis* cells were incubated for 2 h with or not 20 μg of hP6E7 diluted in PBS containing 0.2% of gelatin. Both groups, hP6E7 and control, were incubated with anti-human IgG conjugated with Alexa Fluor 488 in sterile PBS containing 0.2% gelatin, and visualized under a fluorescence microscopy.

The next experiment was to analyze the binding activity of hP6E7 to gp70 on the surface of fungi. To probe this binding, we performed an immunofluorescence assay. Yeast cells of *S. schenckii* and *S. brasiliensis* were incubated with hP6E7 and then labeled with Alexa Fluor 488-conjugated anti-human IgG. As expected, we observed a strong signal on the surface of *S. schenckii* cells (**Figure 3A**). On the other hand, we observed a weak but reliable signal on the *S. brasiliensis* cell wall (**Figure 3B**). This weak fluorescence is due to the low amount of gp70 on the surface of *S. brasiliensis*, as previously demonstrated (Castro et al., 2013).

### hP6E7 Enhances the Phagocytosis of *S. schenckii* by Human Macrophages

**FIGURE 4:**
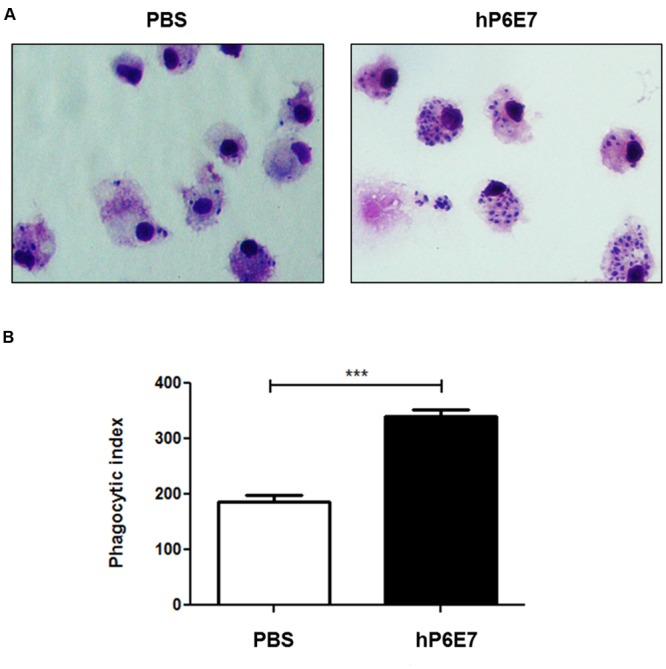
**hP6E7 enhances the phagocytosis against *S. schenckii* by macrophages human cells. (A)** Monocyte-derived macrophages human cells were infected with *S. schenckii* yeast cells opsonized or not by hP6E7 for 5 h. Yeast internalization was counted by optical microscopy. **(B)** Phagocytosis index of yeast cells opsonized with 25 μg of hP6E7 and yeast cells treated with sterile PBS. Data are the means ± SD. ^∗∗∗^*p*< 0.001.

As hP6E7 showed binding activity on the surface of fungus, we evaluated if human monocyte-derived macrophages were able to recognize the Fc human fraction from hP6E7 promoting yeast phagocytosis. Thus, we analyzed the interaction between *S. schenckii* and CD14^+^ human monocyte-derived macrophages. As expected, human monocyte-derived macrophages were able to phagocytose the *S. schenckii* yeast, and opsonized yeast showed a higher phagocytosis ability compared to non-opsonized yeast (**Figure 4**). Our group has already shown that opsonization by mAbP6E7 was able to increase the phagocytosis index in murine macrophages in a concentration-dependent manner and increase NO production (Nascimento et al., 2008). These data demonstrate that hP6E7 is fully functional and is able to recognize the *Sporothrix* sp. antigen, leading to enhanced recognition by phagocytic cells.

### hP6E7 Treatment Decreases the Fungal Burden *In vivo*

**FIGURE 5:**
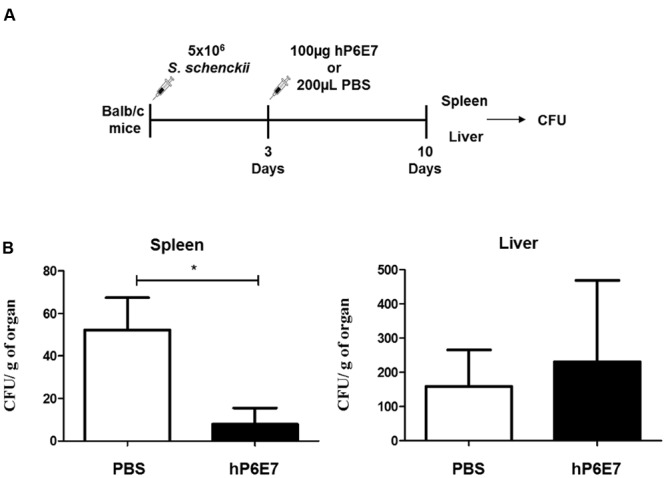
**hP6E7 treatment decreases the fungal burden *in vivo*. (A)** Experimental approach for passive immunization with hP6E7 and fungal burden analysis. **(B)** Fungal burden in the spleen and the liver of infected mice after the treatment with hP6E7 or PBS. Data are the means ± SD. ^∗^*p* < 0.05.

We evaluated if the humanized antibody against gp70 could influence sporotrichosis infection (**Figure 5**). BALB/c mice were treated with hP6E7 after 3 days of *S. schenckii* infection and euthanized after 10 days of infection to evaluate the fungal burden in the spleen and liver (**Figure 5A**). Although hP6E7 has a human Fc fraction, it can be recognized by non-human cells, but with reduced effectivity. The treatment with hP6E7 decreased the fungal burden in the spleen after 10 days of infection compared with the control (**Figure 5B**).

## Discussion

The treatment of sporotrichosis is not always easy, since the drugs of choice to combat sporotrichosis require long periods of treatment, and relapses occur, especially in immunocompromised patients. In some fungal diseases, such as paracoccidioidomycosis (Buissa-Filho et al., 2008) and candidiasis (Moragues et al., 2003) the use of mAbs confers protection. In some case, the use of mAbs modifies the course of infection as observed in experimental infection by *Cryptococcus neoformans* (Rivera et al., 2005). In addition, mAbs against *Cryptococcus neoformans* (Larsen et al., 2005) and *Candida albicans* (Pachl et al., 2006) have been approved for clinical evaluation by FDA. In systemic candidiasis, antifungal antibodies provide novel therapies for use in combination with antifungal drugs (Matthews and Burnie, 2001).

Our group demonstrated that the mAb against gp70 (P6E7) significantly decreased infections of *S. schenckii* and *S. brasiliensis in vivo* (Nascimento et al., 2008; de Almeida et al., 2015). However, the use of the murine monoclonal antibody in clinical practice could induce the production of anti-murine antibodies (HAMA) in patients (Chiu et al., 2011). Therefore, we developed a humanized anti-gp70 antibody as FvFc fragments. The antibody format was chosen for several advantages, as showed in the literature (Holliger and Hudson, 2005). The targeting specificity of the whole mAb is preserved in this fragment; it is poorly immunogenic and has an increased biodistribution and blood clearance properties (Hudson and Souriau, 2003; Holliger and Hudson, 2005).

The process used to complete the humanization of P6E7 replaced the murine components with the amino acid residues of human homologues to reduce the possibility of inducing immunogenicity in patients. Humanized antibodies have been used with success in alternative treatments for several disorders, including cancer, transplant rejection, and infectious diseases (Souriau and Hudson, 2003; Cai et al., 2015; DiGiandomenico and Sellman, 2015). A major problem after the humanization of an antibody is preserving the capacity of the antibody to bind the antigen. After the purification of hP6E7, we tested its capacity to bind gp70. We observed that the humanized antibody was able to bind to gp70, as demonstrated by immunoblotting and immunofluorescent assays. These results indicated that hP6E7 did not lose its affinity and capacity to bind the antigen.

An important mechanism in antibody-mediated protection is the opsonization of the microorganism by the Fc gamma receptor (FCR) (Alvarez et al., 2008). FCR mediates several macrophage functions, including phagocytosis, the stimulation of cytokine secretion, and the triggering of respiratory antibody-dependent cellular cytotoxicity (Clavel et al., 2008; Richards et al., 2008). The results obtained with murine P6E7 showed that the yeast cells with anti-gp70 increased the phagocytic index of murine macrophages. Our results showed that the FvFc fragments of human P6E7 have the ability to bind to gp70 and that the yeast cells of *S. schenckii* with FvFc increase the phagocytic index of monocyte-derived macrophages. Next, our group analyzed the efficiency of hP6E7 to control the disease *in vivo*. Our results showed that treatment with hP6E7 decreased the fungal burden only in the spleen during murine sporotrichosis. Recently, our group also observed the reduction of the fungal burden in spleen, but not in liver after the mAbP6E7 treatment in experimental sporotrichosis by *S. schenckii* and *S. brasiliensis* (de Almeida et al., 2015). As the hP6E7 is a humanized antibody, the Fc gamma receptor (FCR) of mice phagocytes cells can not have a full recognition of the humanized antibody, reducing the antibody efficiency. The spleen is an organ with enough phagocytes cells as macrophages and dendritic cells, being easier to recognize and clear the fungal load in this organ. While in the liver, maybe with more doses the fungal burden could reduce as well. These data show that hP6E7 is efficient in the treatment of sporotrichosis, showing similar results as mAbP6E7 *in vitro* and *in vivo*. Finally, our data suggest that humanized P6E7 could have a therapeutic role in sporotrichosis and may justify further development of the humanized antibody as a biopharmaceutical that could be used in the treatment of fungal infection.

## Author Contributions

JdA, KS, and GK performed the experiments, AM, MdMB, and SdA designed the study and SdA wrote the paper. JdA and KS contributed equally to the work.

## Conflict of Interest Statement

The authors declare that the research was conducted in the absence of any commercial or financial relationships that could be construed as a potential conflict of interest.

## Footnotes

1 http://www.mbio.ncsu.edu/bioedit/bioedit.html

2 http://www.pdb.org
